# The Evaluation of a Mind-Body Intervention (MBT-T) for Stress Reduction in Academic Settings: A Pilot Study

**DOI:** 10.3390/bs10080124

**Published:** 2020-07-30

**Authors:** Mauro Cozzolino, Deborah R. Vivo, Laura Girelli, Pierpaolo Limone, Giovanna Celia

**Affiliations:** 1 Department of Humanities, Philosophy and Education, University of Salerno, 84084 Fisciano, Italy; dvivo@unisa.it (D.R.V.); lgirelli@unisa.it (L.G.); 2 Department of Humanities, Literature, Cultural Heritage, Education Sciences, University of Foggia, 71122 Foggia, Italy; pierpaolo.limone@unifg.it (P.L.); giovanna.celia@unifg.it (G.C.)

**Keywords:** stress management intervention, mind-body therapies, undergraduate students, graduate students, neuroscience research, stress reduction

## Abstract

This study is aimed at evaluating the outcomes of mind-body transformation therapy (MBT-T), previously known as the creative psychosocial genomic healing experience© (CPGHE). The intervention was aimed at reducing the perceived level of stress in two non-clinical groups of students with different educational levels and different expertise in the domain of well-being. Whereas participants from the first group were first-year university students, participants from the second group were students attending a post-graduate program in psychotherapy. All participants (*n* = 159) were exposed to a single session of MBT-T, each group in a separate session. The results of two paired-samples *t*-tests, conducted separately on the two samples, showed that there was a statistically significant reduction in the participants’ perceived level of stress between pre- and post-intervention states in both samples (t_88_ = 5.39, *p* < 0.001; t_53_ = 4.56, *p* < 0.001 respectively). The results, therefore, showed that a single session of MBT-T was beneficial in reducing the perceived level of stress in both first-year university students and students attending a post-graduate program in psychotherapy, regardless of educational level and expertise in the domain of well-being.

## 1. Introduction

Rates of distress are increasing in schools and universities [1]. Current research [2,3] indicates that the recent COVID-19 pandemic, in particular, has significantly worsened mental health issues in students, and there is growing concern regarding the long-term psychological consequences of this outbreak in higher education settings [3,4]. Under normal conditions, undergraduate students witness several important changes related to personal life and education. University students might feel homesick and overwhelmed by academic demands, which might become a major cause of stress for undergraduate students [5,6]. During this transition, students might also experience minor psychological problems related to sleep, eating habits, and concentration [5]. Likewise, more severe psychological problems, such as mental disorders, also share onset within this age range [4], which might explain the high rates of depression, anxiety, and suicidality [2,3,4]. Though a few years older, graduate students, too, are often exposed to significant stress because of the pressure and the challenges posed by post-graduate programs. Moreover, graduate students are more likely to be married and have children and, thus, they might be more exposed to personal and family problems that cause stress in their everyday life. Greater stress may lead to increased anxiety, depression, and suicidality [1]. Among graduate students, we believe that post-graduate medical students and students attending a post-graduate program in psychotherapy can be regarded as a special class because stress-related issues are particularly relevant to their work. Recent studies on post-graduate medical students [2,3] demonstrate that they are under severe stress and that a high level of stress leads to academic underperformance, absenteeism, and a poor quality of life. Moreover, graduate students attending a post-graduate program in psychotherapy, who generally hold a master’s degree in psychology, are expected to be able to command state-of-the art information and other key skills in their field of study, including stress management and well-being. Yet, further pressures arising from this expectation might lead to increased distress and feelings of failure in these students [7,8]. Despite experiencing significant stress, most students do not reach out for professional help [2]. Research indicates that the most important barriers to seeking mental health therapy among young adults include perceived stigma, self-denial of a mental health problem, negative attitudes about treatment, and practical barriers, such as not knowing where to seek help, cost issues, etc. [4,5]. These findings prompt universities and institutions providing undergraduate and post-graduate education and training to ensure that stress management programs are always available and, importantly, that these services are structured in such a way as to engage young people. Mind-body interventions (MBIs) are generally well suited to this purpose because they are often practiced by non-clinical individuals as well, and are often endorsed by celebrities, which steers clear of the stigma usually attached to mental health treatments. Mind-body interventions are designed to enhance the mind’s positive impact on the body [9,10]. They are based on various practices that range from ancient techniques for self-care and well-being (e.g., meditation, yoga, tai chi, etc.) to more modern Western practices (e.g., mindfulness, hypnotherapy, psychological therapies, etc.) [11]. Several studies support the effectiveness of a number of mind-body interventions in reducing stress in university students [12,13,14,15,16]. Moreover, research indicates that the cause of many disease conditions, including inflammatory and neurodegenerative diseases, is a complex interaction between stressful life experiences, the genome, the mind, and behavioral factors [9,17,18,19]. A recent line of research [10,17,20,21,22,23,24] has provided new insights into the pathophysiology of stress-related disorders and it has identified the gene sets involved in a number of biological pathways, including stress response, inflammation, and physical health. These studies describe the genomic and epigenetic pathways of stress, focusing on gene expression changes brought about by mind-body therapies. Despite a number of studies supporting the effectiveness of these interventions in lowering the level of stress, [13,25,26,27,28], improving well-being [29,30,31,32], and academic attainment in student populations [33], they may also present adverse effects and contraindications [34,35,36,37,38], especially for those individuals who are advised against mild to moderate physical exertion. In addition, mind-body therapies often require training that may be challenging to learn, and they are generally time-consuming to perform. In order to avoid some of the issues posed by these interventions, we carried out a study on stress reduction using a novel mind-body technique known as mind-body transformation therapy (MBT-T).

MBT-T—previously known as the creative psychosocial genomic healing experience© (CPGHE) [39,40,41]—is a therapeutic protocol that has been shown to improve therapeutic results without the need for long traditional therapies. From both a theoretical and an empirical perspective, it is derived from the studies of M. H. Erickson and E. Rossi’s Mind–Body Therapy [10,42,43]. The protocol is based on the so-called four-stage creative process [10], which is a very easy to learn procedure, allowing individuals to obtain stress reduction without the need for traditional, complex, and intricate methods.

Although there is extensive literature evaluating stress reduction among university students [44], relatively few studies have evaluated stress reduction techniques in graduate students [25] and we are not aware of many studies on graduate students attending a post-graduate program in psychotherapy. Because we believe that graduate students likely face the same (if not a greater) amount of stress as undergraduates, even if their expertise in stress management is different, we chose to include both groups in our study. Our goal was to evaluate whether the positive effects of the MBT-T intervention on stress in undergraduates would be noted in graduate students as well. Such findings would suggest that MBT-T is a suitable stress reduction intervention in more than one academic setting, with implications for decision-makers regarding the psychological support programs made available for students in such settings.

## 2. Materials and Methods

We used an uncontrolled quasi-experimental design to evaluate the effects of a single MBT-T session on two groups of students sampled by cluster. The first group (Group 1) included *n* = 58 first-year university students, 50% males, mean age = 24.45 (±8.70) years old. The second group (Group 2) consisted of *n* = 101 graduate students attending a post-graduate program in psychotherapy, 22.8% males; mean age = 35.56 (±10.14) years old. Women were overrepresented and the age range was broad, reflecting the typical demographics of the university and post-graduate courses our participants attended.

Overall, 159 students were selected for the study. Participants were all exposed to a single session of MBT-T, each group in a separate session. Stress was measured using the Distress Thermometer (DT) [45,46,47,48,49,50,51,52,53]. The DT is a single-item screening tool that is well validated to be sensitive and specific to the construct of stress [45]. In order to assess change in self-perceived stress, at the beginning of the session, the researcher asked participants to indicate their perceived “initial stress” on an 11-point scale ranging from 0 (*no stress*) to 10 (*maximum stress*) [54]. Once the session was terminated, participants were asked again to indicate their “final stress” on the same scale. Moreover, participants’ feedback about the benefits of CPGHE was also collected. As for the measures, although longer measures for the screening of stress might have been used, we opted for a single-item scale for its brevity and ease of administration. However, we based our choice on studies that validated the DT against other robust measures [45], and confirmed that the single-item DT can be compared with other measures [46]. Besides, a number of validation studies and reviews [45,47,48,49,50] show that the DT has good psychometric properties across countries and cultures. Its sensitivity and specificity, as well as its positive and negative predictive value, are in the range of good overall accuracy (Donovan et al., 2014; Snowden et al., 2014). Moreover, the median scores for these properties are consistent with existing studies written in the English language, whereas different language versions of the DT may have different cut-off scores for clinically significant problems, which is likely due to cultural differences [46,48]. The cut-off score has also been found to change depending on subject characteristics and setting [46,49]. Nonetheless, a cut-off score of four is widely agreed to indicate clinically significant distress [45,46,48]. Thanks to its good psychometric properties, as well as its brevity, the DT was an ideal screening tool to include in a study on stress management with university students.

The stress reduction method we used in this study was MBT-T, previously known as the creative psychosocial genomic healing experience© (CPGHE). The CPGHE is based on the four-stage creative process [10,17], which facilitates positive psychosocial transformations. It presents certain advantages over traditional mind-body methods (i.e., it is very easy to learn, it can be performed in minutes, it can be administered to both single individuals and large groups, it does not require specific premises or tools, and it only demands one researcher). The CPGHE protocol is a four-stage protocol. The first stage is “Focusing Consciousness”, which implies self-awareness of thoughts and feelings. The second stage is “Problem Review”, during which participants assessed the thoughts and feelings from the first stage by focusing on the problem at hand. The third stage is “Problem Solving”, during which participants learned how to cope with the problem arising from the third stage. The final stage is “Self-Care”. Within this stage, participants applied what they had learnt from the third stage to their present situation. Participants were allowed to share their experiences with each other [39]. The CPGHE protocol starts a therapeutic dialogue that may generate new consciousness for a positive exploration of all emotions related to experience, thus reducing acute and/or chronic stress [10] (see Appendix A).

In order to evaluate changes in the level of participants’ stress over time 0 (pre-intervention) and time 1 (post intervention), a paired-samples *t*-test was conducted separately for each sample (undergraduates and post-graduates). The analyses were conducted with IBM SPSS Statistics for Windows, version 23 (IBM Corp., Armonk, New York, NY, USA).

All participants gave their informed consent for inclusion before they participated in the study. Tests were anonymous to ensure the confidentiality and reliability of the data. All procedures in this study were performed in accordance with the ethical standards of the Italian Association of Psychology (AIP) research committee and with the 1964 Helsinki Declaration and its later amendments. No further approval was required.

## 3. Results

**Figure 1:**
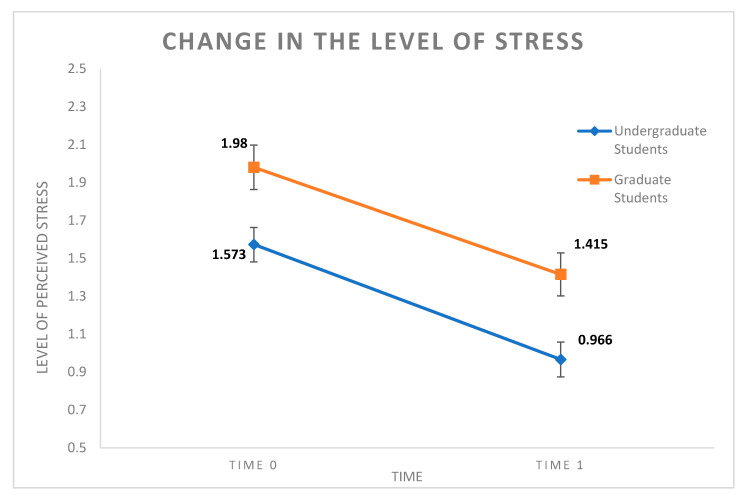
Changes in the level of perceived stress in participants from undergraduate students and graduate students.

We computed two paired-samples *t*-tests to make a pre-test–post-test comparison, one for each sample. The results of the paired-samples *t*-test conducted in undergraduate students showed that the level of post-treatment stress was statistically significantly different and lower than the level of pre-treatment stress (*t* (53) = 4.56, *p* < 0.001), as displayed in Figure 1. Furthermore, the results of the paired-samples *t*-test conducted in graduate students showed that the level of post-treatment stress was statistically significantly different and lower than the level of pre-treatment stress (*t* (88) = 5.39, *p* < 0.001), as is also displayed in Figure 1. In order to test for regression toward the mean, we computed a bivariate correlation between stress scores at t0 and the change scores (calculated by subtracting the time 0 stress scores from the time 1 stress scores) separately for each group of participants. The results of these analyses showed that the correlation was statistically significantly different and negative for both groups (*r* = −0.56, *p* < 0.001; *r* = −0.60, *p* < 0.001 for undergraduate and graduate students, respectively).

## 4. Discussion

The present study described the implementation of MBT-T on stress management to reduce the perceived level of stress in a non-clinical group of university students. We compared the levels of stress before and after a single intervention of MBT-T in a population of both undergraduate students and graduate students attending a post-graduate program in psychotherapy.

The results suggested that a single session of MBT-T could reduce the perceived level of stress among our participants. In particular, our results indicated that CPGHE/MBT-T showed a significant reduction in final stress as measured at the end of the interventions regardless of educational level and previous expertise in the domain of well-being. Our preliminary investigation seems to confirm that MBT-T might be a suitable stress reduction intervention for university students, as well as graduate students attending a post-graduate program in psychotherapy. In being an easy to implement, sustainable, and reproducible intervention, MBT-T might be a suitable approach that students may be willing to use as compared to other types of intervention. Therefore, we believe that if MBT-T were incorporated into undergraduate and post-graduate stress reduction programs, students would probably benefit from it and improve their well-being. The MBT-T presents certain advantages over traditional mind-body methods (i.e., it is very easy to learn, it can be performed in minutes, it can be administered to both individuals and large groups, it does not require specific premises or tools, and it only demands one researcher).

Despite these encouraging findings, we are aware that the uncontrolled quasi-experimental design was a major limitation to our study. Furthermore, the absence of a control group did not allow us to draw definitive conclusions regarding the effects of the intervention and caution must be made when interpreting the presented results. The results of the correlations between stress scores at t0 and the change scores (calculated by subtracting the time 0 stress scores from the time 1 stress scores), showed no regression to the mean. However, future research should address this potential critical threat to internal validity by using a clinical trial with a pre-test–post-test assessment in at least two conditions: experimental and control. Other limitations to our study could have included circadian rhythms, time effects, history effects, local history effects, and test–retest sensitization. Yet, with regard to time effects, the study included a single intervention, which was carried out within the same time slot (from 10 a.m. to 12 p.m.) in both groups. Therefore, neither circadian rhythm nor ultradian cycle effects were relevant. Furthermore, the intervention was carried out before the COVID-19 pandemic, which made history effects and local history effects relatively non-salient. Moreover, we acknowledge that participants may have become sensitized to the measure. Yet, the mechanism of the so-called pre-test effect or pre-test sensitization is typically not investigated or even known [51], therefore, it was difficult for us to reduce this effect in our uncontrolled quasi-experimental study. Finally, we did not include follow-ups because, at the time of this study, our interest was in investigating the technique’s effect after just a single intervention. We acknowledge that the positive results presented may require discounting due to the absence of a control group. Besides, this was a pilot study for a preliminary investigation, and we are planning to carry out a randomized controlled trial to evaluate the technique with more statistical power.

Despite these issues, the present study has a major strength, which is the replication. Although reproducibility is the foundation of science [54,55], direct replication is seldom pursued [54,56]. Furthermore, as one study shows, replication rates are much higher when the original researchers are involved [57]. Our study included replication because it was conducted with two different groups; importantly, it involved the same researchers, so the methods and procedures were carefully mirrored. The results we achieved held true in both settings and populations. Hence, the replication gave greater validity to our findings. In addition, our study could be a springboard for further studies on the effects of MBT-T/CPGHE. This method could be integrated into more structured programs including more sessions (four to eight) over a longer period of time, which would help us understand how the benefits obtained with a single MBT-T intervention can be maintained and/or enhanced over time.

Because stress among university students is a widespread and growing problem [44,52,53], we believe that universities should develop more effective and sustainable stress management programs for students that are based on mind-body therapies. In particular, future research should examine innovative methods like MBT-T. In our view, future studies should investigate the effects of this type of intervention to identify and examine methods to maintain the benefits of stress reduction interventions, as well as their effects on different individuals. For this reason, we hope that researchers will further investigate this field of study, taking into consideration the possible use of MBT-T.

## Appendix A. The Creative Psychosocial Genomic Healing Experience: A Brief Protocol

Introduction

The therapist begins with the following: “It is wonderful to know how our best thoughts and positive feelings can improve health and well-being. Here are a few exercises that will inspire you to explore some interesting questions that can help you solve your problems creatively in your own way.” (Optional facilitation of Stage 1: If needed, the therapist may add a few empathetic statements to clarify whatever concerns and questions some people and the group may have regarding this brief protocol).

The therapist now says: “An important aspect of creative problem solving is to realize how you can explore new life possibilities by looking at things from many points of view at the same time. These creativity exercises will ask you to carefully observe yourself and carefully remember what you are experiencing”.

So, the therapist says: “You can begin by filling out the Distress Thermometer (Pause for a moment). This instrument asks you to record what level of stress you are experiencing right now on a scale of 0 to 10, where 0 is no stress or discomfort and 10 would be the worst stress or discomfort you have ever experienced in your whole life. Go ahead and circle or write in your initial stress level right now on the dotted line.” (Pause for a moment).

Stage 1: Focusing Consciousness

The therapist models the first stage of the creative process, with the palms of the hands about 20 cm apart facing each other at about chest level, and says: “You can begin by looking at your hands like this”.

The therapist asks, “Which hand feels a bit warmer or cooler?” Subjects may sometimes seem puzzled about what this question means. The therapist simply continues, saying “Most people don’t realize how their hands or other parts of their body usually feel slightly warmer or cooler when they really pay attention to it. This is a good exercise to help you become more aware of yourself. It helps to focus your attention and positive feeling about your natural abilities.” After a minute, the therapist adds support and states emphatically, “Notice and remember how warm or cool your hands seem to be.” (Allow another minute for the subject’s inner focus).

The therapist now asks, “Now notice which hand feels stronger or weaker.” After 1 min, the therapist adds support by stating emphatically, “Notice and remember how strong or weak your hand seems to be!” (Allow another minute for the subject’s inner focus).

The therapist asks, “Now notice which hand feels lighter or heavier.” After 1 min, the therapist adds support by stating emphatically, “Notice and remember how light or heavy your hand seems to be!” (Allow another minute for the subject’s inner focus).

Stage 2: Incubation. Problem Review

The therapist asks, “Now let’s explore your imagination. Which hand seems to be you today—and which hand feels more like you as a child?” After 1 min, the therapist adds support by stating emphatically, “Notice and remember which hand seems to be you at your present age and which hand seems to be more like you as a child!” (Allow another minute for the subject’s inner focus).

The therapist now asks, “Which hand represents some problem you would like to solve right now—today in this exercise?” (Therapist pauses for 1 min). “And which hand seems to be the opposite, perhaps holds an answer to your problem?” After 1 min, the therapist adds support by stating emphatically, “Remember which hand represents your problem and which hand seems to hold the opposite—perhaps an answer, even if you do not know what it is yet!” (Allow another minute for the subject’s inner focus).

The therapist states emphatically, “Now let the hand that represents your problem begin to drift down very slowly as you privately review the history, memories, and feelings of your problem from the beginning to the present moment.”

The therapist offers motivational support with these remarks, administered 1 min apart:

• “That’s right! Do you have the courage to allow that hand and arm to drift down a bit … with each memory you find yourself reviewing?”

• “Allow yourself feel only as much of that as you need to and then move on to the next memory that comes up more or less by itself.”

• “That’s right! Let yourself have the courage to continue only as long as you need to to feel everything as fully as you need to privately.”

• “That’s right! While another part of you observes wisely, you learn how to take care of yourself to imagine and create the best possible outcome for yourself.”

When the problem hand finally touches down in the person’s lap, the therapist adds support and offers empathetically, “That’s right! Allow your problem hand drift down to your lap and come to a comfortable rest, wonderful, appreciate your job well done! Remember as much of this Stage 2 of your creative process as you need to build a better future! And now, get ready to move on to the solution of your problem with your other hand. Let your other hand, holding the solutions to your problem, remain up for a moment so you can now turn your full attention to it!”

Stage 3: Illumination and Insight. Problem Solving

The therapist continues saying: “Now allow your other hand to drift down slowly as you explore new possibilities about how to solve your problem today. Allow that hand to begin drifting down slowly as you begin to explore something new. Explore your best hopes and imagination for today and the future, that could be some interesting and wonderful possibilities for problem solving, healing and well-being. Speculate about exciting and fascinating turning points in your life. Create the best of all possible world for yourself. Enjoy your best dreams about yourself!”

The therapist observes the shifts from negativity, stress, sadness, and conflict (of Stage 2) to the more searching expressions of positive expectation in Stage 3 of the creative process that are often punctuated with a slight smile and even a short laugh. He supports these positive shifts with a few warm following remarks, such as these, administered at 1-min intervals:

• “Something pleasantly surprising you can look forward to? What do you really need that is most interesting and important to you?”

• “Simply receive and continue to explore the sources of your strength for dealing successfully with that issue.”

• “Yes, appreciate the value of that as fully as you need to while taking good care of yourself as that hand finally comes to rest in your lap.”

• When the hand finally touches down in the subject’s lap, the therapist states in a supportive manner: ”Remember how real and strong these new positive possibilities and feelings for changing your life for the better are!”

• “Wonderful, really appreciate yourself for a job well done! And now, get ready to move on to the resolution of this issue (concern, problem, or symptom)!”

Stage 4: Reality Testing and Self-Care

The therapist concludes this fourth part with the following remarks, administered at 30-s intervals:

• “When (brief pause for emphasis) a part of you knows it can continue this creative work entirely on your own at appropriate times throughout the day.” (30-s pause)”.

• “And when (brief pause for emphasis) your conscious mind knows it can simply cooperate in helping you recognize when is the right time to tune in and continue this creative work privately on your own.” (30-s pause).

• “Learn how you can explore and practice your new ideas in the real world and give yourself positive prescriptions for taking good care of yourself.” (30-s pause). “You will bring this creative exercise to an end for now so you can stretch and become fully alert.”

The therapist can provide support by stating: “Some of you may wish to share a few of your insights with the group,” or, “All this creative work can remain private within you.”

Optional facilitation of Stage 5: If needed, the therapist may encourage the group to share with supportive remarks such as these:

• “Is there something interesting some of you would like to share about your creative inner work?”

• “What is surprising and unexpected about this that is new to you?”

• “What interesting possibilities are opening up for you to now?”

The therapist asks: “Please complete the Distress Thermometer again by filling in what your stress level is now at the end of your creative exercise.”
